# Renal cell carcinoma with bilateral synchronous adrenal gland metastases: a case report

**DOI:** 10.4076/1757-1626-2-7298

**Published:** 2009-09-09

**Authors:** Georgios E Koutalellis, Evangelos Felekouras, Constantinos Evangelou, Georgios Koritsiadis, Dimitrios Chasiotis, Ioannis Anastasiou

**Affiliations:** 1 Department of Urology, “Laiko” General Hospital, Athens Medical School17 Agiou Thoma str, 11527, Goudi, AthensGreece; 2 Department of Surgery, “Laiko” General Hospital, Athens Medical School17 Agiou Thoma str, 11527, Goudi, AthensGreece; 3 Department of Pathology, “Laiko” General Hospital, Athens Medical School17 Agiou Thoma str, 11527, Goudi, AthensGreece

## Abstract

**Introduction:**

Renal cell carcinoma is characterized by its potential of metastasizing widely and to unusual sites, with the metastases occasionally preceding clinical recognition of the primary tumor. Synchronous bilateral adrenal metastases from renal cell carcinoma, without other metastases, are rare and, to our knowledge, only 17 cases have been published in the literature to date. In general, patients with synchronous bilateral adrenal metastases from renal cell carcinoma have a poor prognosis.

**Case presentation:**

We report a case of right-sided renal cell carcinoma with simultaneous bilateral adrenal metastases in a 58-year-old woman. The primary tumor was localized in the upper and mid pole of the kidney. The diagnosis was established preoperatively by abdominal ultrasound and computed tomography. Surgical treatment consisted of a right radical nephrectomy and bilateral adrenalectomy. Postoperative cortisone acetate replacement was instituted. The pathological findings of the right renal tumor showed clear cell carcinoma and both adrenal tumors showed the same pathology as the right renal tumor. There was no evidence of recurrence after 6 months of follow-up.

**Conclusion:**

Patients with bilateral synchronous adrenal metastases should be considered to have disseminated metastatic disease. However, good performance status, the presence of paraneoplastic syndrome and the alleviation of refractory pain are important reasons make an urologist to consider radical nephrectomy in renal cell carcinoma patient with metastases.

## Introduction

It is well established that the incidence of adrenal metastases in certain types of malignancy, such as carcinomas of the breast, lung and melanoma is high [1]. Renal cell carcinoma (RCC) can metastasize extensively and to unusual sites. Due to the insidious clinical course of many primary RCCs, approximately a third of all patients have metastases at the initial diagnosis. The most common metastatic sites from RCC are the lung, lymph nodes, liver, bone, contralateral kidney and ipsilateral adrenal gland [2]. The incidence of adrenal metastases from RCC is 6-29% in autopsy series [3-5], and one third of this incidence (2-10%) from clinical diagnosis [6-9]. Synchronous bilateral involvement was found to be present at about one fifth of all adrenal metastases [1,10]. Adrenal metastases from RCC are either synchronous or metachronous. In this article, we report a case of right-sided RCC with simultaneous bilateral adrenal metastases, the treatment and prognosis are also discussed.

## Case presentation

**Figure 1.**
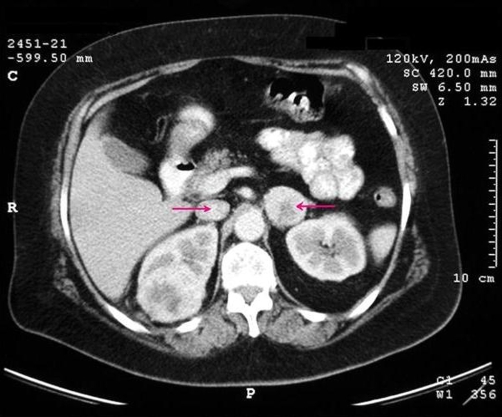
Contrast-enhanced abdominal computed tomography (CT) revealed a 6 cm × 5 cm × 4 cm solid mass at the upper and mid pole of the right kidney that consisted of heterogeneous and enhanced areas and also bilateral involvement of the adrenal glands (arrows).

A 58-year-old Caucasian-Greek woman presented with a dull pain located in the right flank for 4 years. Other symptoms, including hematuria, were not present. The patient had a medical history of spondylolisthesis, osteoporosis, hypertension and hyperlipidemia. The gradual deterioration of pain in the last month before her admission caused her to seek medical attention. The pain initially was attributed to spondylolisthesis and she was prescribed a relative medication. Due to the persistence of pain despite the administration of the specific medication she was submitted to sonography. The ultrasonography revealed a right upper pole renal mass and bilateral adrenal masses. Because of these findings she was admitted for further evaluation. A physical examination revealed a mild tenderness in her right flank. No lymphadenopathy was found. The patient’s blood pressure and the pulse rate were normal. A contrast-enhanced abdominal computed tomography (CT) confirmed the presence of a 6 cm × 5 cm × 4 cm solid mass at the upper and mid pole of the right kidney with heterogeneous and enhanced areas and bilateral involvement of the adrenal glands (Figure 1). A chest x-ray, chest CT scan, and bone scan were all negative for metastases. Laboratory tests revealed no abnormalities. Endocrinological evaluation demonstrated normal serum and urinary levels of aldosterone, cortisol and 17-hydroxysteroids. The diagnosis of right renal cell carcinoma with bilateral adrenal metastases was established.

Right radical nephrectomy was performed through a bilateral subcostal incision (Chevron). The contents of Gerota’s fascia were removed en bloc, followed by a total left adrenalectomy. Thorough exploration of the abdominal cavity did not disclose any other metastatic focus.

**Figure 2.**
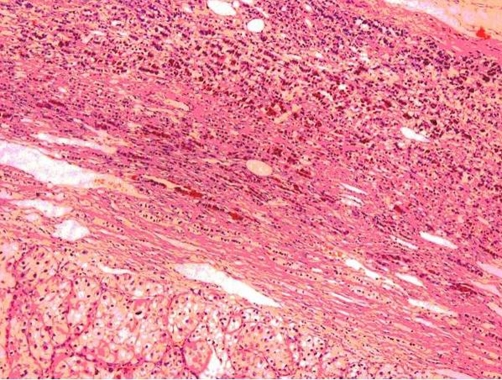
Histological section of clear RCC metastasis in the left adrenal gland. Only a thin rim of adrenal tissue is preserved (H & E stain, 200X).

Histological examination of the resected specimens revealed a solid tumor with golden-yellow appearance, relatively well circumscribed and measuring 5.6 × 4.8 × 3.5 cm in the upper half of the right kidney. Almost the entire left adrenal gland, measuring 6.2 × 3.1 × 2.7 cm, was occupied by a brownish compact tumor while only a thin rim of adrenal tissue was preserved. A same mass, with a maximum diameter of 1.9 cm, was also observed in the right adrenal gland. Microscopic examination of the renal and both adrenal gland tumors revealed the diagnosis of clear cell renal carcinoma, Grade3 and pT3 according to the TNM classification system (Figure 2). The carcinoma invaded and focally disrupted the renal pelvis. Invasion of the perinephric fat to a limit extend as well as of various vessels by neoplastic cells was also observed.

Convalescence was uneventful. The dosage of cortisone was gradually tapered to 12.5 mg per day orally. Serum cortisol and electrolytes were normal during the follow-up period. She was discharged 10 days after the operation.

The patient is alive with no evidence of disease recurrence or metastatic disease after six months postoperatively treated with adjuvant chemotherapy.

## Discussion

Adrenals involvements are usually silent because metastatic lesions fail to destroy enough tissue to produce clinical signs or symptoms of insufficiency. However, adrenal function is usually maintained even though bilateral adrenal glands are affected by malignancy. There is a higher incidence of upper pole primary renal tumors than either mid or lower pole renal tumors in patients with adrenal metastases [11]. Patients with adrenal metastases tend to have large primary renal tumors that nearly encompass the entire kidney [11]. Adrenal metastases result from direct extension of the renal tumor, lymphatic spread from involved retroperitoneal nodes, retrograde venous embolization and local vascular spread through vessels within Gerota’s fascia [12].

The presence of ipsilateral adrenal involvement appears to portend a poor prognosis. These patients have high-stage primary tumors (T3 or greater). Antonelli et al. [10] concluded that ipsilateral adrenal sparing procedure is only safe during surgery for organ-confined RCC of ≤4 cm, while the location of cancer according to preoperative CT findings cannot fully predict the risk of ipsilateral metastases. Moreover, any suspicious contralateral adrenal mass should be explored because there is a high probability of removing a metastasis. In an another study, authors recommended ipsilateral adrenalectomy in conjunction with radical nephrectomy for RCC being indicated in case of large, upper pole extracapsular lesions; even then, it does not affect prognosis [13].

Patients with RCC and a single contralateral adrenal metastasis should be considered as having a solitary metastasis. They should undergo radical nephrectomy and contralateral adrenalectomy. Ipsilateral adrenalectomy should be performed if there is suspicion of metastasis [14]. These patients appear to have better survival than those with bilateral adrenal metastases.

Patients with bilateral synchronous adrenal metastases from RCC should be considered to have disseminated metastatic disease. There are only 18 cases of bilateral adrenal metastases from RCC, without other metastases, that have been reported in the literature, including our case. The follow-up duration is less than 4 years. Despite prophylactic treatment after the operation with chemotherapy and immunotherapy the prognosis remains poor.

An alternative treatment option in cases of RCC with bilateral adrenal metastases is partial adrenalectomy, sparing the normal adrenal tissue [15]. This procedure results in the preservation of the adrenal function while decreasing the tumor burden. In such a manner, steroid replacement and its side effects such as gastritis, hypertension, hypokalemia and Addisional crises during stressful periods can be avoided.

However, the goal of any surgical or medical procedure should be to improve the quality of life and duration of survival. Radical nephrectomy in RCC patients with metastases may be considered in the presence of paraneoplastic syndrome provided the patient is of good performance status. Surgical intervention may also be indicated for palliative reasons. In our case, the patient hadn’t paraneoplastic syndrome, was in good performance status, and the surgical intervention performed for the alleviation of severe, refractory pain.

## Conclusions

In our opinion, some patients with bilateral adrenal metastases from RCC and without metastases in other site may be benefited by radical operation, as regards their overall survival, because the reduction of the tumor burden may enhance response rates to postoperative adjuvant therapy.

## Abbreviations

CT: computed tomography
RCC: renal cell carcinoma
